# Exploring the Components of Multicultural Competence among Pre-Service Teacher Students in Thailand: An Approach Utilizing Confirmatory Factor Analysis

**DOI:** 10.3390/ejihpe14090164

**Published:** 2024-08-29

**Authors:** Bovornpot Choompunuch, Khanika Kamdee, Prakittiya Taksino

**Affiliations:** 1 Faculty of Education, Khon Kaen University, Khon Kaen 40002, Thailand; bovornpot.psy@gmail.com; 2 Faculty of Humanities and Social Sciences, Khon Kaen University, Khon Kaen 40002, Thailand; khanka@kku.ac.th

**Keywords:** multicultural competence, cultural awareness, cultural knowledge, personal skills, pre-service teachers, confirmatory factor analysis

## Abstract

The aim of this study is to examine the components of multicultural competence among pre-service teacher students in Thailand and to develop and assess the reliability of a model of multicultural competence for pre-service teacher students in Thailand. Multistage stratified random sampling was utilized to recruit 600 pre-service teacher students from undergraduate education programs at the Faculty of Education in Thailand. Data were collected through self-report questionnaires. The data analysis employed descriptive statistics and second-order confirmatory factor analysis (CFA). The findings indicate that multicultural competence among pre-service teacher students comprises three components: cultural awareness, cultural knowledge, and personal skills. Furthermore, this study identified that the model of multicultural competence among pre-service teacher students demonstrated good fit indices for the modified model (χ^2^ = 30.902, df = 21, *p*-value = 0.0753, χ^2^/df = 1.472; root mean square error of approximation (RMSEA) = 0.028; standardized root means square residual (SRMR) = 0.013; Tucker–Lewis index (TLI) = 0.996; comparative fit index (CFI) = 0.999). Based on these findings, effective teaching in diverse environments necessitates a thorough understanding of multicultural competence.

## 1. Introduction

Multicultural competence is a crucial construct in teacher education, particularly for pre-service teachers preparing to engage with increasingly diverse classrooms [1,2]. Multicultural competence involves the ability to understand, communicate with, and effectively interact with people across cultures. It includes awareness of one’s own cultural worldview, knowledge of different cultural practices and worldviews, and cross-cultural skills [3,4,5,6,7,8,9,10]. This competence is vital for fostering an inclusive and effective educational environment.

Multicultural competence is defined by scholars as the combination of self-reflection on personal biases and worldviews, teaching efficacy, and an understanding of various cultural backgrounds [1,2]. These components are essential for pre-service teachers to cultivate effective teaching practices and promote inclusive education [5,6]. Self-awareness and an understanding of diverse populations are foundational to teaching effectiveness [7].

Self-efficacy beliefs, which refer to an individual’s confidence in their ability to perform specific tasks, play a pivotal role in shaping pre-service teachers’ readiness for diverse classrooms [11,12]. Studies have shown that self-efficacy influences teaching practices and can be enhanced through professional experiences [13]. Therefore, evaluating multicultural competence must include an assessment of pre-service teachers’ confidence in instructing diverse student populations [14].

Inclusive education is another critical aspect of multicultural competence. Research indicates that pre-service teachers need a comprehensive understanding of inclusive practices and opportunities to reflect on their attitudes towards inclusion to foster positive outlooks [15]. Promoting inclusive practices and addressing biases are crucial for enabling pre-service teachers to engage effectively with diverse students.

The development of instruments like the Multicultural Counseling Inventory (MCI) highlights the importance of assessing multicultural competencies in educational settings [16,17]. Utilizing validated tools can help evaluate pre-service teachers’ multicultural competencies and guide interventions to enhance their effectiveness in diverse classrooms.

In Thailand, efforts are ongoing to align higher education with the country’s development goals, emphasizing the need to equip pre-service teachers with the skills to navigate multicultural classrooms effectively [18]. The increasing diversity in Thai educational settings underscores the importance of research on multicultural competence among pre-service teacher students [19]. Understanding the components of multicultural competence is vital for designing teacher education programs that meet the needs of diverse student populations.

A primary challenge in promoting multicultural competence is the lack of awareness regarding systemic factors influencing mental health and career development in diverse classrooms [20]. Training that increases awareness of these factors can provide pre-service teachers with a deeper understanding of the complexities involved in teaching in multicultural environments. Supervisors play a crucial role in this process by facilitating discussions on diversity within the supervisory relationship [21]. Effective mentorship and guidance are essential for helping pre-service teachers navigate multicultural challenges.

Multicultural competence is closely linked to self-efficacy beliefs and a commitment to social justice among counseling psychology trainees [22]. Enhancing self-efficacy and fostering a commitment to social justice can improve the overall multicultural competence of pre-service teachers, enabling them to create inclusive and supportive learning environments. This study employs a confirmatory factor analysis approach to methodically examine the components underpinning multicultural competence [23]. By analyzing the interrelations among variables such as self-efficacy, inclusive practices, and cultural awareness, this study aims to achieve a nuanced understanding of how these elements collectively shape multicultural competence among pre-service teacher students in Thailand.

Investigating the components of multicultural competence among pre-service teacher students in Thailand through confirmatory factor analysis is crucial for enhancing teacher education programs. By addressing challenges, leveraging supervisory guidance, and considering the influences of self-efficacy and social justice commitment, educators can better prepare pre-service teachers to meet the diverse needs of multicultural educational settings.

Given the significance of multicultural competence, this research focuses on identifying its components among pre-service teacher students. Following the identification of these components, a training program was developed to enhance the multicultural competence of pre-service teachers. The primary objectives of this study are to identify and validate the key components of multicultural competence among pre-service teacher students in Thailand, examine the relationships between these components and their relative contributions to overall multicultural competence, and provide insights for the development of targeted interventions and training programs to enhance multicultural competence in teacher education. By determining and confirming the essential elements of multicultural competence, analyzing how these components interrelate and contribute to overall competence, and offering recommendations for effective training, this study aims to equip pre-service teachers with the skills to effectively teach in diverse classrooms and impart knowledge on enhancing multicultural competence in learners, ultimately preparing them for entry into a culturally diverse society.

## 2. Materials and Methods

The cross-sectional design of this study allowed for an in-depth examination of the components of multicultural competence among pre-service teacher students in Thailand. This approach also enabled the development and reliability assessment of a multicultural competence model specifically tailored to this cohort. The researchers synthesized documents and studies on multicultural competence (MCC) from various educators to identify relevant components and indicators [3,4,5,6,7,8,9,10]. This methodology aligns with the findings of Mueller [24], Hu and Bentler [25], and Kanchanawasi [26], who emphasize the effectiveness of confirmatory factor analysis in establishing construct validity. As a result of this synthesis, three components with 12 indicators were selected for investigation.

**Figure 1:**
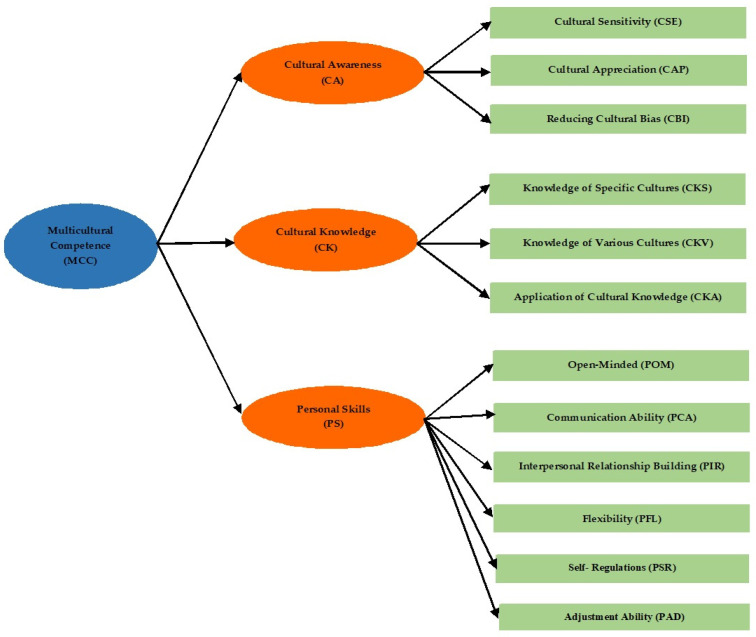
Conceptual framework for studying the components of multicultural competence for pre-service teacher students.

Component 1, cultural awareness (CA), comprises 3 indicators: cultural sensitivity (CSE), cultural appreciation (CAP), and reduction in cultural bias (CBI). Component 2, cultural knowledge (CK), encompasses 3 indicators: knowledge of specific cultures (CKS), knowledge of diverse cultures (CKV), and application of cultural knowledge (CKA). Component 3, personal skills (PS), includes 6 indicators: open-mindedness (POM), communication ability (PCA), interpersonal relationship building (PIR), flexibility (PFL), self-regulation (PSR), and adjustment ability (PAD). The researchers propose a conceptual framework for studying the components of multicultural competence among pre-service teacher students in this research, as depicted in Figure 1.

The sampling cohort utilized to investigate these components consisted of undergraduate students enrolled in the Faculty of Education in Thailand during the academic year 2022, procured through multi-stage sampling. Confirmatory factor analysis was employed in this study to ascertain the validity of the measurement model. Therefore, a sizable sample size was imperative, and the distribution within the population should approximate a normal curve. Following the guidelines proposed by Hair et al. [27], which advocate for a sample size ranging from 5 to 20 times the number of parameters for confirmatory factor analysis, a total of 600 students were deemed appropriate and adequate for this investigation. The procedural steps undertaken were as follows:

Step 1: Categorize universities based on their regional affiliations, employing the regional division criteria stipulated by the Department of Provincial Administration, Ministry of Interior, which delineates four regions: Central, North, Northeast, and South.

Step 2: Within each region, designate higher education institutions under the purview of the Ministry of Higher Education, Science, Research, and Innovation (MHESI) as the sampling units (inclusive of autonomous and public universities). Employing simple random sampling, randomly select one university from each region, resulting in a total of four universities, with one representing each region.

Step 3: Employ stratified random sampling to randomly select pre-service teacher students from each university, categorized by their year of study (2nd to 4th year), with 50 students sampled per year level. First-year students are excluded from the sample as they have recently commenced their program and lack professional teaching experience in educational settings. This yields a sample size of 150 students per university, culminating in a total research sample of 600 students.

Step 4: Gather data utilizing accidental sampling to obtain the predetermined number outlined by the sampling methodology employed in this study.

### 2.1. Research Procedure

**Figure 2:**
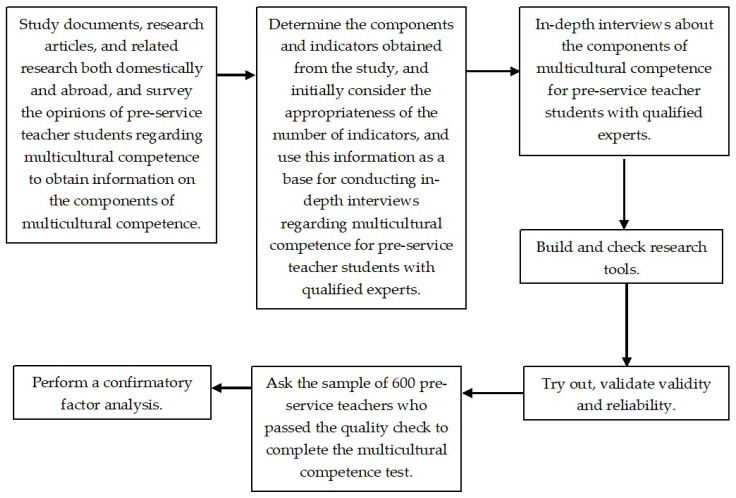
Steps for studying the components of multicultural competence for pre-service teacher students.

In conducting this study, the researchers executed the following steps, as depicted in Figure 2:

1. Literature Review and Survey
  1.1 We engaged in an exhaustive review of literature, encompassing documents, research articles, and related studies both domestically and internationally.
  1.2 We administered surveys to pre-service teacher students to understand their perspectives on multicultural competence. The survey took approximately 30–40 min to complete. The purpose of sending questionnaires to pre-service teachers in the early phases of the investigation was to gather preliminary data that would inform the development of our multicultural competence model. This initial data collection was essential for understanding the baseline levels of multicultural competence among pre-service teachers and identifying any immediate gaps or needs in their training.
2. Component and Indicator Determination
  2.1 We identified the components and indicators derived from the aforementioned study.
  2.2 We initially assessed the appropriateness of the number of indicators.
  2.3 We utilized this foundational information to conduct in-depth interviews concerning multicultural competence for pre-service teacher students with recognized experts in the field.

Our approach was grounded in several well-established theoretical frameworks on multicultural competence. We drew on the works of scholars such as Banks [3], who emphasizes the importance of cultural awareness and knowledge in education, and Sue and Sue [4], who highlight the significance of personal skills in multicultural interactions. These frameworks provided a robust foundation for selecting the indicators of multicultural competence.

3. In-Depth Expert Interviews
  3.1 We conducted comprehensive interviews concerning the components of multicultural competence for pre-service teacher students with a panel of qualified experts comprising 5 experts from the faculty or lecturers of the Faculty of Education, 5 experts specializing in cultural psychology, and 5 experts proficient in measurements and research pertaining to the components and observed variables of multicultural competence in pre-service teachers. These experts were selected based on their extensive experience and contributions to the field. The interviews were conducted over several sessions, each lasting approximately one hour. The experts were asked to provide their insights on the essential components of multicultural competence and to review our preliminary indicators. The data collected from these interviews were analyzed thematically, and the findings were used to validate and refine our model. The expert input ensured that our indicators were both theoretically sound and practically relevant.
4. Tool Development and Validation
  4.1 We developed and validate research instruments based on insights garnered from literature reviews, surveys, and expert interviews. It comprised 104 items, with each item answered on a 5-point rating scale to assess multicultural competence in pre-service teachers, with scores ranging from ‘strongly agree’ (5) to ‘strongly disagree’ (1), following the approach outlined in [28]. The mean scores were divided into five levels: lowest (1.00–1.50), low (1.51–2.50), moderate (2.51–3.50), high (3.51–4.50), and highest (3.51–5.00) [29]. The content validity was appraised by a panel of 5 experts, ensuring a content validity index (CVI) of no less than 0.8 [30], as well as evaluating language suitability. Each item achieved a CVI score of 1.00 for this study.
5. Pilot Testing
  5.1 We executed a pilot study to evaluate the efficacy and reliability of the developed research instruments. The multicultural competence test for pre-service teachers underwent validation, achieving a Cronbach’s Alpha Coefficient (α) of 0.972 in this examination.
6. Confirmatory Factor Analysis
  6.1 We performed confirmatory factor analysis on the data obtained from the pilot study to validate the research instruments.
7. Administration of Multicultural Competence Test for Pre-service Teachers
  7.1 We administered the multicultural competence test to a subset of pre-service teachers who had passed the quality assurance checks.
  7.2 We implemented this assessment with a larger sample, as outlined (n = 600), to ensure the statistical significance and reliability of the findings.

Following the data collection from the questionnaires and expert interviews, we applied CFA to validate the proposed model. This statistical method allowed us to test the fit of our model and ensure that the selected indicators reliably measured the components of multicultural competence.

### 2.2. Data Analyses

The Statistical Package for the Social Sciences (SPSS) version 25.0 and M-Plus software version 8.4 were employed for data analyses. Descriptive statistics were utilized to assess demographic data and all variables. Pearson’s correlation and Spearman’s rank correlation coefficient were employed to explore the relationships among the measured variables. Additionally, confirmatory factor analysis (CFA) was conducted to establish the confirmatory model of multicultural competence among pre-service teacher students in Thailand. Before commencing data analysis, all pertinent assumptions were verified. The goodness of fit of the hypothesized model was evaluated based on various criteria, including (a) a chi-square test (χ^2^, *p* < 0.05); (b) the normed chi-square (χ^2^/df) with a preferred value of <2.0; (c) the root mean square error of approximation (RMSEA), with values approaching 0.0 considered favorable; (d) the comparative fit index (CFI), with values approaching 1.0 deemed optimal; (e) the standardized root mean square residual (SRMR), with values approaching 0.0 indicative of a good fit; and (f) the Tucker–Lewis index (TLI), with values approaching 1.0 signaling a satisfactory fit [31]. A significance level of *p* < 0.05 was adopted for all analyses.

## 3. Results

The analysis of personal factors among the 600 respondents of the multicultural competence test for pre-service teacher students revealed the following demographics: The majority were female (63.00%), with an average age of 20.62 years, ranging from 18 to 23 years. Most respondents identified as Buddhists (86.80%). Academically, the most common field of study was Thai language (15.20%), and the largest proportion of participants were in their fourth year (34.00%). Participants were evenly distributed across educational institutions in different regions of Thailand, with 150 individuals (25.00%) from each of the North, Central, Northeast, and South regions. In terms of international exposure, the majority of respondents (71.30%) had no prior experience of traveling abroad (see Table 1).

In this study, the researchers analyzed the scores from the multicultural competence test for pre-service teacher students, focusing on competence and component measurements, including mean values and standard deviations.

The investigation revealed that the overall multicultural competence among pre-service teacher students was notably high, with a mean score of 4.09 (*S.D*. = 0.52). When examining the individual components, they each exhibited high mean scores. Cultural awareness had the highest mean score (Mean = 4.26, *S.D*. = 0.50), followed by cultural knowledge (Mean = 4.09, *S.D*. = 0.55) and personal skills (Mean = 3.92, *S.D*. = 0.61).

Further analysis of the sub-components within each principal category showed that within the cultural awareness domain, reducing cultural bias achieved the highest mean score (Mean = 4.35, *S.D*. = 0.56). Within the cultural knowledge domain, knowledge of specific cultures had the highest mean score (Mean = 4.14, *S.D*. = 0.56), while within the personal skills domain, adjustment ability had the highest mean score (Mean = 4.23, *S.D*. = 0.61) (see Table 2).

The multicultural competence scores for pre-service teacher students consist of 12 sub-components. They exhibit a correlation coefficient ranging from 0.560 to 0.788 and demonstrate statistically significant correlations at the 0.01 level, as depicted in Table 3.

The results of the confirmatory factor analysis of multicultural competence for pre-service teacher students showed that all components had factor loadings (*β*) ranging from 0.805 to 0.998, all statistically significant at the 0.01 level. The first-order components included three main facets: cultural awareness (CA), cultural knowledge (CK), and personal skills (PS), each with robust sub-components. Notably, within cultural awareness (CA), the highest factor loading was for cultural appreciation (CAP). Within cultural knowledge (CK), knowledge of various cultures (CKV) had the highest factor loading, and within personal skills (PS), communication ability (PCA) had the highest factor loading.

For the second-order components, cultural knowledge (CK) had the highest factor loading (β = 0.998), followed by cultural awareness (CA) (*β* = 0.924) and personal skills (PS) (*β* = 0.831). Additionally, the analysis indicated that each component had a substantial prediction coefficient (R^2^). Cultural knowledge (CK) had the highest R^2^ value (R^2^ = 0.996), indicating its ability to explain 99.6% of the variance in multicultural competence for pre-service teacher students (see Table 4).

The model fit indices for the second-order confirmatory factor analysis of cultural competence among pre-service teacher students, considering Carmines and McIver’s criteria [31], revealed a Chi-square value of 30.902 with a corresponding *p*-value of 0.0753, which is not statistically significant at the 0.05 level. Other fit indices fell within acceptable ranges, with a Relative Chi-square (χ^2^/df) of 1.472, below the threshold of 2.0, a comparative fit index (CFI) of 0.999, and a Tucker–Lewis index (TLI) of 0.996, both approaching 1.0. Additionally, the Standardized root mean square residual (SRMR) was 0.013, and the root mean square error of approximation (RMSEA) was 0.028, both nearing 0.0. These analytical findings suggest that the second-order confirmatory factor model of cultural competence among pre-service teacher students is well aligned with empirical data, as illustrated in Table 5.

**Figure 3:**
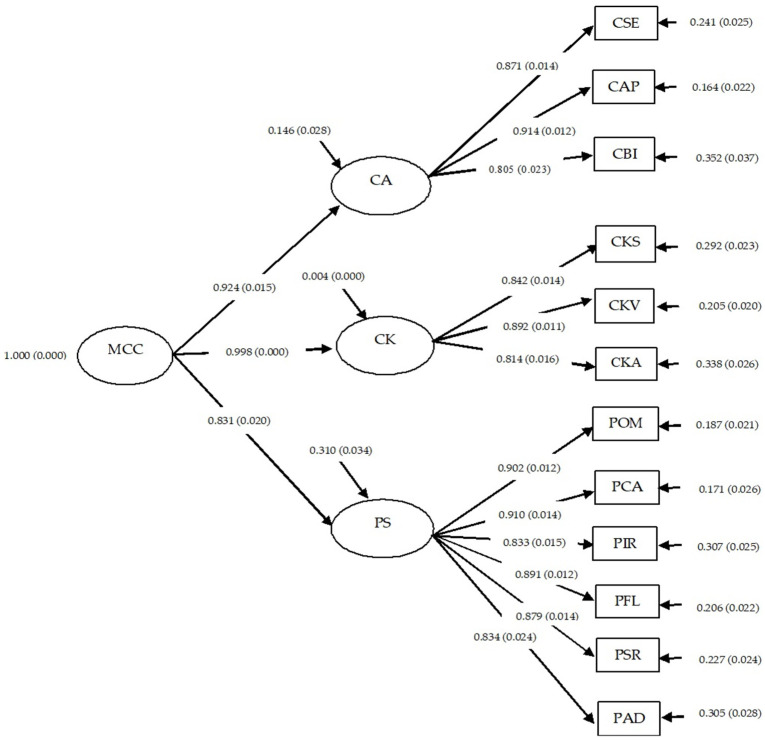
Second-order confirmatory factor analysis model of the components of multicultural competence for pre-service teacher students. χ^2^ = 30.902, df = 21, *p*-value = 0.0753, RMSEA = 0.028, SRMR = 0.013, CFI = 0.999, TLI = 0.996.

To clearly present the cultural competence model for pre-service teacher students, the researchers introduced the second-order confirmatory factor model of cultural competence for pre-service teacher students, as illustrated in Figure 3.

## 4. Discussion

The exploration of multicultural competence components among pre-service teacher students in Thailand has provided crucial insights with significant implications for educational practice and policy formulation. Through confirmatory factor analysis, three primary components emerged: cultural awareness, cultural knowledge, and personal skills. These components are foundational for pre-service teachers as they prepare them to navigate the complexities of increasingly diverse classroom settings effectively.

The notable standardized factor loadings observed across all components attest to the robustness and reliability of the proposed model. Particularly significant is the prominence of cultural awareness, which has the highest mean score. This indicates strong recognition among pre-service teachers of the importance of understanding and sensitively engaging with cultural diversity. This finding aligns with scholarly discourse, such as the work of Spanierman et al. [5], which highlights the critical role of self-awareness and a comprehensive understanding of diverse populations in fostering effective teaching practices.

Previous research has consistently demonstrated the positive impact of enhancing cultural awareness among educators on teaching practices and student outcomes [32]. For instance, Moore [33] accentuated the significance of fostering special educators’ cultural awareness through collaborative reflective professional learning communities, showcasing the transformative influence of cultural awareness in fostering inclusive learning environments. This underscores the critical necessity of promoting cultural awareness among educators to engender supportive and inclusive educational settings. Furthermore, the sphere of cultural awareness extends beyond realms such as healthcare and education, permeating various facets of society, including legal systems and community dynamics. Azizah [34] delved into the challenges of promoting gender equality within patriarchal cultural contexts, emphasizing the pivotal role of raising awareness to address gender justice issues. Similarly, Desi and Prayoga [35] underscored the significance of augmenting community legal awareness concerning the implementation of Islamic Sharia law, thereby underscoring the transformative potential of education in fostering cultural understanding and promoting enduring societal values. These multifaceted studies collectively underscore the intricate interplay between cultural awareness and its profound impact on shaping social structures and norms.

The substantial significance of personal skills, encompassing attributes such as open-mindedness, effective communication, and adaptability, resonates with research findings articulated by Miller and Sendrowitz [22]. Their work underscores the indispensable nature of such skills in establishing inclusive and supportive learning environments conducive to fostering student success. Furthermore, the evident high level of cultural knowledge discerned from the data underscores the profound understanding pre-service teachers possess regarding diverse cultural backgrounds. This acumen is pivotal for implementing culturally responsive pedagogies, as emphasized by seminal works such as that of Gay and Howard [36]. Exploratory studies, such as the research conducted by Constantine and Ladany [37], have delved into the intricate relationship between self-reported multicultural counseling competence scales and underlying social desirability attitudes. Such inquiries shed invaluable light on the nuanced interplay between personal attributes and professional competencies. A comprehensive understanding of how personal skills influence multicultural case conceptualization abilities is paramount for enhancing counselors’ efficacy in addressing the multifaceted needs of diverse clientele. Moreover, the pivotal role of personal skills in counselor training and professional development has been a focal point within the field of psychology. A seminal national survey conducted by Holcomb-McCoy and Myers [38] unearthed a discernible correlation between ethnicity and perceived levels of multicultural competence among counselors. This underscores the profound influence of personal characteristics and cultural awareness in shaping counselors’ capacities to deliver culturally competent care. Such insights underscore the indispensable nature of ongoing efforts to cultivate and harness personal attributes conducive to promoting culturally sensitive and effective professional practice within counseling contexts.

An integral aspect of this study is the examination of multicultural competence. Previous scholarly inquiries, such as the study conducted by Holcomb-McCoy and Myers [38], have delved deeply into the realm of multicultural competence and its intersection with counselor training. Their findings revealed a discernible correlation between ethnicity and perceived levels of multicultural competence among professionals in counseling contexts. This observation underscores the profound influence of personal background factors on the manifestation of multicultural competencies within professional domains. Furthermore, it underscores the paramount importance of introspection and the implementation of recommendations within the domain of multicultural competency research, as underscored by Pope-Davis et al. [39]. Such scholarly endeavors underscore the enduring commitment within the academic community to enhance multicultural competence through the cultivation of reflective practices and the strategic deployment of interventions. These initiatives signify ongoing efforts aimed at fortifying the professional capacity of individuals to navigate the complexities of diverse cultural landscapes with sensitivity, efficacy, and cultural humility.

Similarly, within the domain of education, Wang [40] undertook a comprehensive analysis of teacher education programs and educator perceptions to bolster teachers’ multicultural competence within the context of Chinese ethnic minority education. The study elucidated the formidable challenges posed by the multicultural characteristics inherent in students from ethnic minorities, thereby accentuating the imperative of furnishing teachers with the requisite skills to adeptly address the diverse educational needs that ensue. Furthermore, Mena and Rogers [41] embarked on an exploration of factors associated with multicultural teaching competence, with particular emphasis on the pivotal role played by social justice orientation and exposure to multicultural environments in shaping educators’ competencies. This multifaceted examination underscores the nuanced interplay of diverse factors influencing professionals’ ability to effectively engage with heterogeneous populations within educational settings.

The data gleaned from the sample group evince notable diversity across various demographic dimensions, including gender, age, and fields of study. However, despite this heterogeneity, the sample exhibits pronounced homogeneity in terms of ethnicity (Thai) and religious affiliation (Buddhism). The unique cultural, social, and educational dynamics of Thailand, including its specific demographic composition and socio-political climate, significantly influence the development and assessment of multicultural competence. Consequently, the model validated in this study may not directly translate to educational settings with different cultural backgrounds, such as those in Western countries with diverse gender distributions and predominant Christian influences. Additionally, variations in educational systems and policies further complicate the generalizability of our findings. Therefore, caution is advised when extrapolating our results to other contexts, and further research is necessary to adapt and validate the model in different geographical and cultural settings. This will ensure the development of effective, context-specific training programs that enhance multicultural competence globally. This observation prompts consideration of the necessity for initiatives aimed at broadening pre-service teachers’ exposure to and engagement with a more expansive array of cultural and religious contexts. Such endeavors hold promise for fostering the development of their multicultural competence [42].

The outcomes derived from the confirmatory factor analysis affirm the presence of robust multicultural competence among pre-service teacher students in Thailand, aligning closely with empirical evidence. This concordance with empirical data echoes the assertions of Mueller [24], Hu and Bentler [25], and Kanchanawasi [26], who have underscored the utility of confirmatory factor analysis in providing evidence of construct validity. Furthermore, our findings are congruent with the research insights of Chunpen [43], whose investigation into the cross-cultural competence model of teachers identified three principal components: cultural awareness, knowledge of culture, and personal skills. Similarly, Chiu [44] and Puhy [45] expounded upon a similar tripartite model of cross-cultural competence among educators, comprising cultural awareness, cultural knowledge, and personal skills. These scholarly contributions collectively underscore the multifaceted nature of cross-cultural competence among educators, elucidating key domains essential for effective intercultural engagement within educational contexts.

The finding that a significant majority of our sample had not traveled abroad highlights an important consideration regarding the development of cultural awareness. While international travel can indeed broaden one’s exposure to diverse cultures and enhance cultural competence, it is not the sole pathway to achieving this awareness. Cultural competence can be effectively developed through local multicultural experiences, such as engaging with diverse communities within one’s own country, participating in cultural exchange programs, and attending multicultural events. Additionally, education plays a crucial role; incorporating multicultural curricula and fostering inclusive classroom environments can significantly enhance cultural awareness [44]. Reflective practices, such as self-assessment, critical reflection on personal biases, and discussions on cultural diversity, are also essential strategies [45]. These alternatives provide robust avenues for developing cultural competence, emphasizing that meaningful engagement and education, rather than travel alone, are key to fostering an inclusive and culturally aware mindset.

Drawing from the insights garnered in this study, several recommendations emerge. First, we must implement comprehensive training programs addressing all facets of multicultural competence—cultural awareness, cultural knowledge, and personal skills—to adequately equip pre-service teachers for the complexities of multicultural classroom environments. We should continuously evaluate and refine teacher education curricula to reflect the dynamic nature of cultural competence, ensuring alignment with current best practices and research findings. Second, we must cultivate an educational milieu conducive to self-reflection, ongoing learning, and meaningful engagement with diverse cultures to enhance pre-service teachers’ competence and confidence. We should employ the developed multicultural competence test as a diagnostic tool to pinpoint areas of strength and areas requiring further development among pre-service teachers. Third, we must advocate for initiatives and policies fostering multicultural awareness and training within teacher education programs, recognizing the pivotal role of educators in shaping the social and cultural competencies of future generations. Additionally, we should endorse international collaboration and exchange programs to expose pre-service teachers to diverse cultural contexts, thereby enriching their understanding and experiential knowledge. Finally, we must undertake longitudinal studies to trace the evolution of multicultural competencies over time and evaluate the enduring impact of targeted training initiatives. Moreover, we should expand research methodologies to encompass qualitative approaches, offering deeper insights into how pre-service teachers integrate multicultural competence into their instructional practices.

While our study provides valuable insights into the components of multicultural competence among pre-service teacher students in Thailand, several limitations should be considered when applying these findings to different contexts. First, the model developed and validated in this study is tailored to the Thai educational context, which has unique cultural, social, and educational dynamics. Extrapolating these findings to other countries with different cultural backgrounds, such as those with a predominantly Western or Christian context, should be carried out with caution. Second, the demographic composition of our sample, including gender distribution and cultural backgrounds, may differ significantly from those in other contexts. For instance, our sample may not reflect the gender balance or the ethnic and religious diversity found in other educational settings, which could influence the generalizability of our results. Third, the structure and policies of the Thai educational system are distinct from those in other countries. Differences in teacher education programs, curricula, and pedagogical approaches can impact the relevance and applicability of our model to other educational systems. Fourth, the socio-political climate in Thailand, including attitudes towards multiculturalism and diversity, may differ from that in other regions. These contextual factors play a critical role in shaping teachers’ multicultural competence and may not be directly transferable to other socio-political environments. Finally, this study is geographically limited to Thailand, and while it provides in-depth insights into this specific context, broader generalizations should be made cautiously. Our findings are most relevant to similar Southeast Asian contexts but may require adaptation for use in significantly different geographical areas. By acknowledging these limitations, we emphasize the need for further research to validate and adapt our model of multicultural competence in diverse educational settings globally. Future studies should consider these contextual differences to enhance the applicability and effectiveness of multicultural competence training programs in various contexts.

## 5. Conclusions

The study’s findings provide a clear understanding that multicultural competence among pre-service teacher students in Thailand is defined by three key components: cultural awareness, cultural knowledge and understanding, and personal skills. Each of these components shows statistical significance and strong alignment with empirical data. These findings significantly contribute to the existing literature on multicultural competence in education, offering a model that can serve as a benchmark for similar research and practical applications in various contexts. The confirmatory factor analysis model developed in this study underscores the importance of the three identified components in cultivating multicultural competence. Each component is statistically significant, highlighting their essential roles. The second-order confirmatory factor analysis further validates the model’s effectiveness in assessing the multicultural competence of student teachers, demonstrating its utility as a robust evaluative tool. This model provides educators and policymakers with a reliable framework for assessing and enhancing the multicultural competencies of pre-service teachers. It supports the notion that comprehensive multicultural education for pre-service teachers requires not only the acquisition of cultural awareness and knowledge but also the development of personal skills essential for effective cross-cultural interactions.

## Figures and Tables

**Table 1:** The sociodemographic status of the sample who responded to the multicultural competence test for pre-service teacher students.

Demographic Characteristics	Number (Person)	Percentage (%)
Gender		
Male	167	27.80
Female	378	63.00
Alternative Gender	55	9.20
Age		
19 years	94	15.70
20 years	183	30.50
21 years	202	33.70
22 years	101	16.80
23 years	20	3.30
Religion		
Buddhism	521	86.80
Christianity	12	2.00
Islam	67	11.20
Field of Study		
Thai Language/Teaching Thai	91	15.20
English Language/Teaching English	86	14.30
Chinese Language	10	1.70
Mathematics/Teaching Mathematics	71	11.80
Science/Teaching Science	76	12.70
Early Childhood Education	27	4.50
Year of Study (year)		
2nd	200	33.30
3rd	196	32.70
4th	204	34.00
Region of Institution		
North	150	25.00
Central	150	25.00
Northeast	150	25.00
South	150	25.00
Experience Traveling Abroad		
Yes	172	28.70
No	428	71.30

**Table 2:** Means and standard deviation of the multicultural competence test for pre-service teacher students.

Component	Mean (M)	Standard Deviation (SD)
**Cultural Awareness (CA)**	4.26	0.50
1. Cultural sensitivity	4.21	0.54
2. Valuing culture	4.22	0.53
3. Reducing cultural bias	4.35	0.56
**Cultural Knowledge (CK)**	4.09	0.55
1. Specific cultural knowledge	4.14	0.56
2. Diverse cultural knowledge	4.08	0.59
3. Applying cultural knowledge	4.04	0.66
**Personal Skills (PS)**	3.92	0.61
1. Open-mindedness	3.87	0.72
2. Communication ability	3.91	0.66
3. Building interpersonal relationships	3.75	0.75
4. Flexibility	3.87	0.72
5. Self-regulation	3.90	0.70
6. Adaptability	4.23	0.61

**Table 3:** Correlation coefficients of multicultural competency components for pre-service teacher students.

Components		Components of Multicultural Competence for Pre-Service Teacher Students											
		Cultural Awareness (CA)				Cultural Knowledge (CK)				Personal Skills (PS)			
		CSE	CAP	CBI	CKS	CKV	CKA	POM	PCA	PIR	PFL	PSR	PAD
**Cultural awareness (CA)**	**CSE**	1											
	**CAP**	0.798 **	1										
	**CBI**	0.711 **	0.775 **	1									
**Cultural knowledge (CK)**	**CKS**	0.706 **	0.730 **	0.736 **	1								
	**CKV**	0.705 **	0.728 **	0.660 **	0.755 **	1							
	**CKA**	0.633 **	0.624 **	0.569 **	0.670 **	0.764 **	1						
**Personal skills (PS)**	**POM**	0.624 **	0.607 **	0.543 **	0.678 **	0.764 **	0.746 **	1					
	**PCA**	0.625 **	0.660 **	0.562 **	0.688 **	0.745 **	0.703 **	0.727 **	1				
	**PIR**	0.560 **	0.563 **	0.474 **	0.625 **	0.680 **	0.669 **	0.756 **	0.788 **	1			
	**PFL**	0.597 **	0.604 **	0.539 **	0.643 **	0.731 **	0.690 **	0.708 **	0.706 **	0.792 **	1		
	**PSR**	0.562 **	0.612 **	0.528 **	0.648 **	0.734 **	0.671 **	0.766 **	0.711 **	0.731 **	0.772 **	1	
	**PAD**	0.660 **	0.691 **	0.643 **	0.642 **	0.705 **	0.617 **	0.598 **	0.642 **	0.565 **	0.653 **	0.675 **	1

Note: ** *p* < 0.01; CSE = cultural sensitivity; CAP = cultural appreciation; CBI = reducing cultural bias; CKS = knowledge of specific cultures; CKV = knowledge of various cultures; CKA = application of cultural knowledge; POM = open-mindedness; PCA = communication ability; PIR = interpersonal relationship building; PFL = flexibility; PSR = self-regulation; and PAD = adjustment ability.

**Table 4:** Factor loadings (β), statistical significance tests (t), and prediction coefficients (R^2^) of the confirmatory factor analysis.

Cultural Competence Components	Component Weight Matrix			Factor Score	R^2^
	β	SE	t		
**Component 1**					
**Cultural Awareness (CA)**					
1. Cultural sensitivity (CSE)	0.871	0.014	61.617 **	0.241	0.759
2. Appreciation of cultural values (CAP)	0.914	0.012	74.866 **	0.164	0.836
3. Reduction in cultural bias (CBI)	0.805	0.023	35.348 **	0.352	0.648
**Cultural Knowledge (CK)**					
1. Specific cultural understanding (CKS)	0.842	0.014	61.829 **	0.292	0.708
2. Diverse cultural understanding (CKV)	0.891	0.012	77.399 **	0.205	0.795
3. Application of cultural knowledge (CKA)	0.813	0.016	49.906 **	0.338	0.662
**Personal Skills (PS)**					
1. Open-mindedness (POM)	0.901	0.012	72.466 **	0.189	0.811
2. Communication ability (PCA)	0.911	0.015	60.262 **	0.169	0.831
3. Interpersonal relationship building (PIR)	0.834	0.017	49.696 **	0.304	0.696
4. Flexibility (PFL)	0.892	0.014	64.399 **	0.204	0.796
5. Self-regulation (PSR)	0.881	0.017	52.558 **	0.223	0.777
6. Adaptability (PAD)	0.835	0.025	33.945 **	0.303	0.697
**Component 2**					
1. Cultural awareness (CA)	0.924	0.015	61.280 **		0.854
2. Cultural knowledge (CK)	0.998	0.000	91.106 **		0.996
3. Personal skills (PS)	0.831	0.020	40.609 **		0.690

Notes: ** *p* < 0.01.

**Table 5:** Test indices for the second-order confirmatory factor analysis model of the multicultural competence for pre-service teacher students.

	Criterion	Criteria for Evaluation
Chi-square (χ^2^)	30.902	Not statistically significant
*p*-value	0.0753	<0.05
Degrees of Freedom (df)	21	-
Relative Chi-square (χ^2^/df)	1.472	<2.0
Comparative fit index (CFI)	0.999	Value approaching 1.0
Tucker–Lewis index (TLI)	0.996	Value approaching 1.0
Standardized root mean square residual (SRMR)	0.013	Value approaching 0.0
Root mean square error of approximation (RMSEA)	0.028	Value approaching 0.0

## Data Availability

The data sets of this study are not publicly available as the information could compromise the research participants’ privacy. However, the data may be shared upon request to the authors.
